# Comparison of IRES and F2A-Based Locus-Specific Multicistronic Expression in Stable Mouse Lines

**DOI:** 10.1371/journal.pone.0028885

**Published:** 2011-12-21

**Authors:** Hsiao Yun Chan, Sivakamasundari, Xing Xing, Petra Kraus, Sook Peng Yap, Patricia Ng, Siew Lan Lim, Thomas Lufkin

**Affiliations:** Stem Cell and Developmental Biology, Genome Institute of Singapore, Singapore, Singapore; Brigham and Women's Hospital, United States of America

## Abstract

Efficient and stoichiometric expression of genes concatenated by bi- or multi-cistronic vectors has become an invaluable tool not only in basic biology to track and visualize proteins *in vivo*, but also for vaccine development and in the clinics for gene therapy. To adequately compare, *in vivo*, the effectiveness of two of the currently popular co-expression strategies - the internal ribosome entry site (IRES) derived from the picornavirus and the 2A peptide from the foot-and-mouth disease virus (FDMV) (F2A), we analyzed two locus-specific knock-in mouse lines co-expressing *SRY-box containing gene 9* (*Sox9*) and *enhanced green fluorescent protein* (*EGFP*) linked by the IRES (*Sox9^IRES-EGFP^*) or the F2A (*Sox9^F2A-EGFP^*) sequence. Both the constructs expressed Sox9 and EGFP proteins in the appropriate Sox9 expression domains, with the IRES construct expressing reduced levels of EGFP compared to that of the F2A. The latter, on the other hand, produced about 42.2% Sox9-EGFP fusion protein, reflecting an inefficient ribosome ‘skipping’ mechanism. To investigate if the discrepancy in the ‘skipping’ process was locus-dependent, we further analyzed the *FLAG_3_-Bapx1^F2A-EGFP^* mouse line and found similar levels of fusion protein being produced. To assess if EGFP was hindering the ‘skipping’ mechanism, we examined another mouse line co-expressing *Bagpipe homeobox gene 1 homolog* (*Bapx1*), *Cre recombinase* and *EGFP* (*Bapx1^F2A-Cre-F2A-EGFP^*). While the ‘skipping’ was highly efficient between *Bapx1* and *Cre*, the ‘skipping’ between *Cre* and *EGFP* was highly inefficient. We have thus demonstrated in our comparison study that the efficient and close to equivalent expression of genes linked by F2A is achievable in stable mouse lines, but the EGFP reporter may cause undesirable inhibition of the ‘skipping’ at the F2A sequence. Hence, the use of other reporter genes should be explored when utilizing F2A peptides.

## Introduction

Advancement in cloning technologies has led to the construction of polycistronic vectors, which have been used for the co-expression of multiple proteins from a single promoter for vaccine production (antigen expression) [1], *in vivo* stable antibody production [2], multimeric protein expression (e.g. T-cell receptor) [3], [4], cell tracking and enrichment [5], [6] and even for reprogramming human somatic cells to induced pluripotent stem (iPS) cells [7].

Two of the most popular strategies that are employed to co-express multiple genes in a single mRNA are the use of viral internal ribosome entry site (IRES) sequence (internal cap-independent initiation) and 2A oligopeptide sequences (cis-acting hydrolase elements “CHYSEL”) among many others such as fusion proteins, post-translational enzymatic processing, alternative splicing, internal promoters and reinitiation [8].

Over the past two decades, IRES elements of viral and cellular origins have been identified and the former (e.g. IRES from encephalomyocarditis (EMCV) or poliovirus (PV)) has been traditionally utilized in bi- and poly-cistronic vectors [9]. When the IRES element is included between two open reading frames (ORFs), initiation of translation occurs by the canonical 5′- m^7^GpppN cap-dependent mechanism in the first ORF and a cap-independent mechanism in the second ORF downstream of the IRES element [10]. Although the exact processes mediating the internal entry of ribosomes is unknown, it is largely believed that the complex secondary structure adopted by the IRES-encoded RNA sequence and/or the IRES-*trans* acting factors (ITAFs) are responsible for the function of IRES [10], [11]. Unlike the reinitiation mechanism which is highly inefficient and the alternative splicing method that is difficult to regulate for application in multicistronic vectors, IRES enables successful co-expression of the coupled genes. More importantly, it does not give rise to fusion proteins which may adversely affect the activity of the proteins [8]. Conversely, IRES is known for yielding lower amounts of protein from the downstream genes and this phenomenon is believed to exacerbate with increasing tandems; an effect observed in a cell-type specific manner [8], [10], [12]. It is postulated that such alterations in expression levels are due to the varied requirements of the ITAFs for different IRESs and the availability of different ITAFs in diverse cell types. Also, its large size (∼500 bp or more) often makes it an unattractive option for use in viral vectors which have a limited packaging capacity [8], [10].

Subsequent to the discovery of the viral IRES elements another set of viral components, the 2A peptides, was identified in the foot-and-mouth disease virus (FMDV) and later in other genera of *Picornaviridae* family like the cardioviruses [13]. Also termed as CHYSEL, these peptides ‘self-cleave’ their primary 2A/2B polyproteins by a ribosomal ‘skipping’ mechanism which entails inhibition of the peptide bond formation between the C-terminal glycine residue of the 2A peptide and the N-terminal proline residue of the 2B peptide [14]. Mutation analyses and alignment studies on 2A/2B peptide sequences in various viruses such as the EMCV, FMDV and Theiler's murine encephalitis virus (TMEV) have revealed an essential, consensus motif -DxExNPG↓P- for the ‘skipping’ function (↓ represents the position of ‘skipping’) [15]. This motif was also identified in type C rotaviruses, insect viruses, repeated sequences in *Trypanosoma* species and in the alpha-glucosiduronase protein of the hyperthermophilic bacterium *Thermotoga maritima*. Of these, the bacterial sequence alone could not give rise to discrete proteins from the concatenated genes, indicating that the presence of the motif alone is insufficient for the ‘skipping’ mechanism [15]. Also, N-terminal extension of the FMDV 2A (F2A) polyprotein, from 19 amino acids to 33 amino acids, increased the ‘skipping’ efficiency from 90% to >99%, proving that the upstream sequences are also instrumental to the 2A function [15].

This ‘skipping’ property of the 2A peptides has since been exploited to construct multicistronic vectors and has recently gained popularity over the IRES elements. When inserted between two genes, after a ‘skip’ at the glycine-proline residues of 2A/2B, the ribosome continues to translate the second gene thus producing two discrete proteins. Their compactness (19–33 amino acids) in length and their ability to allow the concatenated genes to be translated at equivalent levels both *in vitro* and *in vivo*, have served to complement the flaws in the IRES-based vectors. Nonetheless, there are variations in the ‘skipping’ efficiency of the 2A peptides derived from different viruses (e.g. T2A of Thosea asignavirus, P2A of Porcine teschovirus-1, F2A or E2A of Equine rhinitis A virus) [16]. Moreover, differences in the ‘skipping’ efficiencies of the same 2A peptide in *in vitro* translation, *in vivo* cell culture (including variation in different cell types) or animal models, have been observed in numerous studies [2], [4], [6], [17], [18], [19].

As no extensive investigation on the ‘skipping’ effectiveness of these 2A peptides or a comparison of IRES vs. 2A in bi- or multi-cistronic vectors has been performed in the mouse model in an inheritable manner, we sought to assess if these aspects were dependent on the context of the genes being linked in the constructs.

In this study, by using the transcription factors (TFs) SRY-box containing gene 9 (Sox9) and Bagpipe homeobox gene 1 homolog (Bapx1) as examples, we have shown that both IRES and 2A mediate co-expression of these TFs with enhanced green fluorescent protein (EGFP) and Cre recombinase when concatenated in bi- and tri-cistronic vectors. As EGFP is one of the most commonly used reporter proteins in mouse transgenics owing to its enhanced photostability and strong fluorescence intensity [20], [21], [22], it is necessary to study its compatibility with the IRES- and F2A-based vector systems. Through the analysis of six locus-specific EGFP knock-in mouse lines that we have generated – *Sox9^IRES-EGFP^*, *Sox9^F2A-EGFP^*, *FLAG_3_-Bapx1^F2A-EGFP^*, *Bapx1^IRES-Cre-IRES-EGFP^*, *Bapx1^F2A-Cre-F2A-EGFP^* and *Bapx1^EGFP^* (hereafter these mouse lines will be denoted by their abbreviated forms as summarized in Table 1), we have demonstrated that both the IRES and F2A reliably co-expressed the concatenated proteins. However, unlike the F2A, the IRES consistently yielded a lower expression level of the downstream protein regardless of the locus. We have also shown that the EGFP reporter protein, but not the Cre or the TFs, may substantially hinder the ‘skipping’ function of F2A in the polyproteins. Hence, it is vital for *in vivo* studies requiring tagging of endogenous genes with reporter proteins in multicistronic vectors to either explore the use of other reporter proteins when F2A is used or to assess other 2A peptides for their efficacy when an EGFP reporter protein is utilized.

**Table 1:** Mouse genotype nomenclature.

Mouse Genotype	Abbreviated Name	Alternative Name	*Neo*	*EGFP*	*Cre*
*Sox9^IRES-EGFP^*	*Sox9^IE^*	*Sox9^tm1.Tlu^*	No	Yes	No
*Sox9^F2A-EGFP^*	*Sox9^FE^*	*Sox9^tm2.Tlu^*	No	Yes	No
*FLAG_3_-Bapx1^F2A-EGFP^*	*FLAG_3_-Bapx1^FE^*	*Bapx1^tm2.Tlu^*	No	Yes	No
*Bapx1^IRES-Cre-IRES-EGFP^*	*Bapx1^ICIE^*	*Bapx1^tm3.Tlu^*	No	Yes	Yes
*Bapx1^F2A-Cre-F2A-EGFP^*	*Bapx1^FCFE^*	*Bapx1^tm4.Tlu^*	No	Yes	Yes
*Bapx1^EGFP^*	*Bapx1^EGFP^*	*Bapx1^tm5.Tlu^*	No	Yes	No

*Neo* – *neomycin resistance gene*; *EGFP* – *enhanced green fluorescent protein*; *Cre* – *Cre recombinase.*

## Results

### *Sox9^IE^* vs. *Sox9^FE^* mice

**Figure 1:**
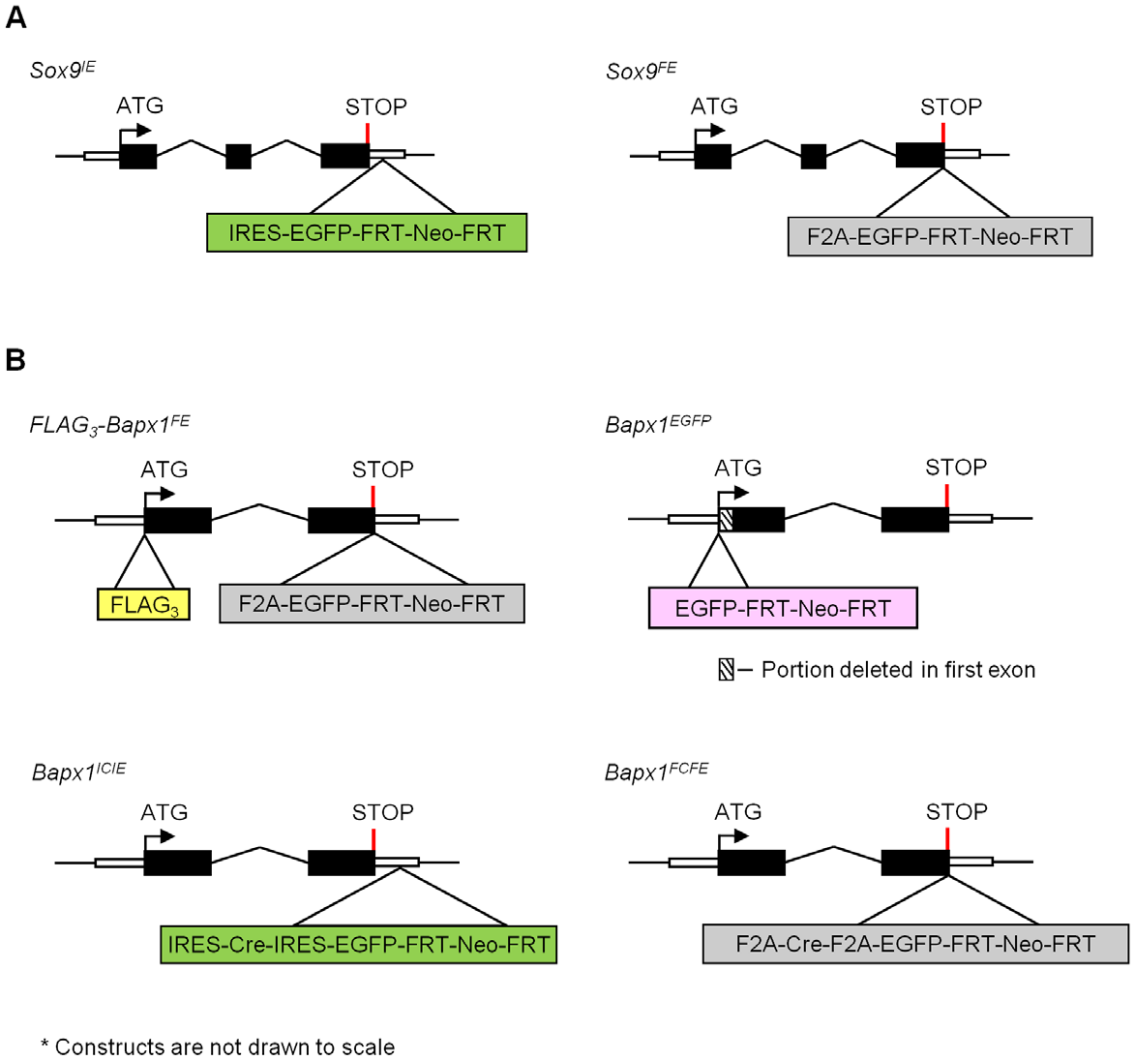
General gene targeting strategy for the *Sox9* and *Bapx1* loci. All exons are depicted as black boxes; intronic sequences as black solid lines and untranslated regions (UTRs) as open boxes. (A) The *IRES-EGFP-FRT-Neo-FRT* and the *F2A-EGFP-FRT-Neo-FRT* cassettes were inserted in the 3′UTR or just before the stop codon of the *Sox9* locus for *Sox9^IE^* (left) and *Sox9^FE^* (right) respectively. (B) Top left: *FLAG_3_-Bapx1^FE^*, triple tandem repeats of *FLAG*-epitope tag and *F2A-EGFP-FRT-Neo-FRT* were inserted at the N-terminus of *Bapx1* just after the ATG translational start site and the C-terminus just before the stop codon respectively; top right: *Bapx1^EGFP^, EGFP-FRT-Neo-FRT* cassette was inserted just after the ATG translational start site of *Bapx1* with a deletion of the first 30 amino acids of *Bapx1* exon1, bottom left: *Bapx1^ICIE^*, *IRES-Cre-IRES-EGFP-FRT-Neo-FRT* cassette inserted in the 3′UTR at *Bapx1* locus; bottom right: *Bapx1^FCFE^*, *F2A-Cre-F2A-EGFP-FRT-Neo-FRT* cassette was inserted just before the stop codon. *F2A, 2A peptide sequence from foot-and-mouth disease virus*; *EGFP, enhanced green fluorescent protein*; *FRT, Flp recombinase recognition target*; *Neo, neomycin*; *IRES, internal ribosome entry site sequence*; *Cre, Cre recombinase*.

To compare the co-expression efficiency of the widely-used EMCV IRES with the emerging alternative – 2A peptide from FMDV (F2A) – in gene-targeted mice, we inserted either *IRES-EGFP* or *F2A-EGFP* at the 3′ end of the *Sox9* locus (as shown in Fig. 1A). Sox9, a HMG-box-containing transcription factor critical in driving chondrogenesis in embryonic skeletal development [23], [24], [25], was chosen for this comparison because of its high expression level in the mouse embryos, starting at E8.5 [26], facilitating visualization and experimentation. To eliminate any possible adverse effects of the 2A peptide residue (23 amino acids) at the C-terminal end of Sox9, a furin protease recognition site (RAKR) was included right after Sox9 which trims the residual 2A peptide from the upstream protein. This would leave only two extra amino acids (arginine and alanine) at the C-terminus of Sox9 [2]. In addition, a Gly-Ser-Gly (GSG) spacer was added just before the F2A sequence in order to enhance the translational ‘skipping’ [3], [27].

### EGFP efficacy assessed by fluorescence intensity and flow cytometry

**Figure 2:**
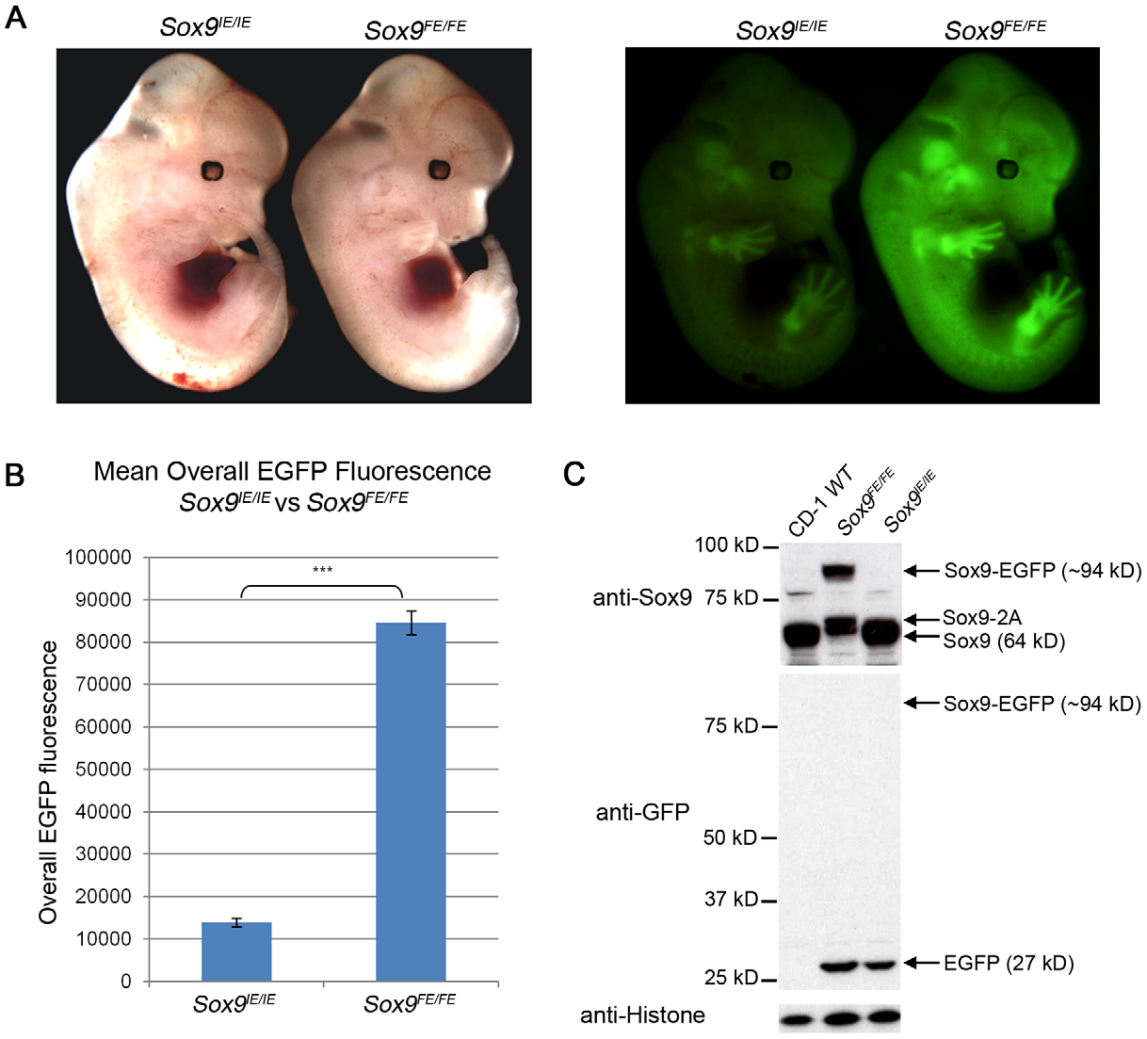
Comparison of E12.5 *Sox9^IE/IE^* and *Sox9^FE/FE^* embryos based on EGFP intensity and Western blotting. (A) Left panel: white light images of E12.5 *Sox9^IE/IE^* and *Sox9^FE/FE^* embryos; right panel: same embryos imaged by fluorescence microscopy showed a higher EGFP fluorescence in the *Sox9^FE/FE^* embryo compared to the *Sox9^IE/IE^* embryo, with EGFP expression in *Sox9*-specific domains in both. (B) Mean overall EGFP fluorescence of E12.5 *Sox9^IE/IE^* (13880.04±1015.55; n = 8) and *Sox9^FE/FE^* (84579.48±2822.09; n = 4) embryos analyzed by flow cytometry. Overall EGFP fluorescence was calculated by multiplying the MFI and percentage of EGFP-positive cells. Two-tailed Student's T test, ***p<0.0001. (C) Western blot analysis of E12.5 *Sox9^IE/IE^* and *Sox9^FE/FE^* embryo lysates, resolved on SDS-polyacrylamide gel electrophoresis (SDS-PAGE) gels and immunoblotted with anti-Sox9 (upper panel) and anti-GFP (middle panel) antibodies. In the upper panel, Sox9-EGFP fusion protein and Sox9-2A (2A - residual 23 amino acids of F2A) was only detected in the *Sox9^FE/FE^* embryo lysate, but not in the CD-1 WT or *Sox9^IE/IE^* embryo lysates. Middle panel: Sox9-EGFP fusion protein was not detected in the *Sox9^FE/FE^* embryos but EGFP was detected in both the *Sox9^IE/IE^* and *Sox9^FE/FE^* lysates at predicted molecular weight. CD-1 WT lysate served as negative control for EGFP. Bottom panel: immunoblotting with anti-histone antibody showed equal loading. WT – wild-type.

E12.5 embryos were harvested from the two mouse lines on the same day and examined under a fluorescence microscope. While both the homozygous *Sox9^IE/IE^* and *Sox9^FE/FE^* mouse embryos expressed EGFP appropriately in the Sox9 expression domains such as the vertebral column and limbs [26], the overall intensity of fluorescence was distinctly higher in the *Sox9^FE/FE^* mouse embryos (Fig. 2A). The embryos were dissociated into single cells and a further analysis of EGFP expression by FACSARIA Cell Sorter substantiated the intensities. The mean overall EGFP fluorescence of the *Sox9^FE/FE^* embryos (84579.48±2822.09; n = 4) was 6.09-fold higher (p<0.0001) than that of the *Sox9^IE/IE^* embryos (13880.04±1015.55; n = 8) (Fig. 2B, Table 2 and S1).

**Table 2:** Fold change of the overall EGFP fluorescence and the p-value.

X	Y	Fold change of mean overall EGFP fluorescence(^X^/_Y_)	p-value
*Sox9^FE/FE^*	*Sox9^IE/IE^*	6.09	4.70E-11
*Bapx1^EGFP/EGFP^*	*Bapx1^FCFE/FCFE^*	1.46	2.92E-02
*Bapx1^EGFP/EGFP^*	*Bapx1^ICIE/ICIE^*	3.11	2.33E-07
*Bapx1^FCFE/FCFE^*	*Bapx1^ICIE/ICIE^*	2.13	3.27E-03

EGFP – Enhanced green fluorescence protein; Overall EGFP fluorescence was calculated by multiplying MFI and percentage of EGFP^+^ cells.

### Stoichiometric protein expression assessed by Western blot

To study the *in vivo* stoichiometric protein expression of Sox9 and EGFP, cell lysates from E12.5 *Sox9^IE/IE^* and *Sox9^FE/FE^* embryos were probed by Western blot (WB) using anti-GFP and anti-Sox9 antibodies (Fig. 2C). The amount of EGFP in the IRES embryos was slightly less than that in the F2A embryos in spite of the equal loading of both the cell lysates as detected by the anti-histone antibody (Fig. 2C and Table S3). Based on our densitometric analysis, there was also 42.2% Sox9-EGFP fusion protein detected by the anti-Sox9 antibody in the F2A embryos (Fig. 2C and Table S3). However, this fusion protein was not detected by the anti-GFP antibody, possibly owing to its lower sensitivity compared to the anti-Sox9 antibody. We also identified a band slightly higher than the predicted 64 kD size for Sox9 (Sox9-2A) in the F2A embryo lysate which indicated that the RAKR protease site failed to induce cleavage of the residual 2A peptide from the upstream Sox9 (Fig. 2C).

### ‘Skipping’ inefficiency is not locus-dependent as revealed by *FLAG_3_-Bapx1^FE^* mice

**Figure 3:**
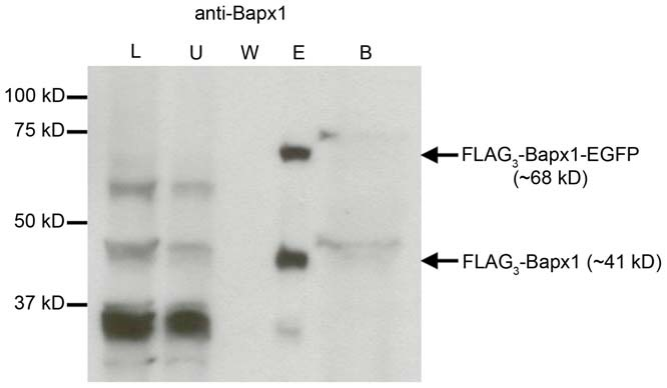
Western blot analysis of F2A efficiency at the *Bapx1* locus. Cell lysates from ten E12.5 *FLAG_3_-Bapx1^FE^* embryos were affinity purified using anti-FLAG antibody-conjugated beads. Protein fractions from total lysate, first and last wash, eluate and boiled beads were subjected to SDS-PAGE. Immunoblotting by anti-Bapx1 antibody detected FLAG_3_-Bapx1-EGFP fusion protein and discrete FLAG_3_-Bapx1 protein only in the eluate. L, total cell lysate; U, unbound protein (first wash); W, wash (last wash); E, eluate; B, boiled beads.

To investigate if the ‘skipping’ efficiency was dependent on the upstream gene, we analyzed another mouse line in which *F2A-EGFP* was inserted at the C-terminus of *Bapx1* (Fig. 1B). Murine *Bapx1* (*Nkx3.2*) is a member of the NK homeobox gene family and due to prior knowledge of its confined and specific expression domains in cartilaginous tissues of the limbs and vertebral column during embryogenesis [28], [29], it was selected as an alternative upstream gene to supplement the study. Unlike Sox9, Bapx1 protein is not as abundantly expressed during embryonic development [29] and cannot be easily detected on WB. Therefore, a triple FLAG tag (24 bp) was added at the N-terminus of *Bapx1* (*FLAG_3_-Bapx1^FE^*, Fig. 1B) to allow enrichment of Bapx1 protein by affinity purification. We performed one round of purification of cell lysate from ten *FLAG_3_-Bapx1^FE^* mouse embryos with anti-FLAG antibodies prior to WB detection with anti-Bapx1 antibody. We noted a similar degree of ‘skipping’ deficiency in the *FLAG_3_-Bapx1^FE^* mice as revealed by the amount of Bapx1-EGFP fusion protein (49.4%) detected (Table S3). Hence, despite targeting a different locus, we still observed a high percentage of fusion protein (Fig. 3).

### *Bapx1^ICIE^* vs. *Bapx1^FCFE^* mice

**Figure 4:**
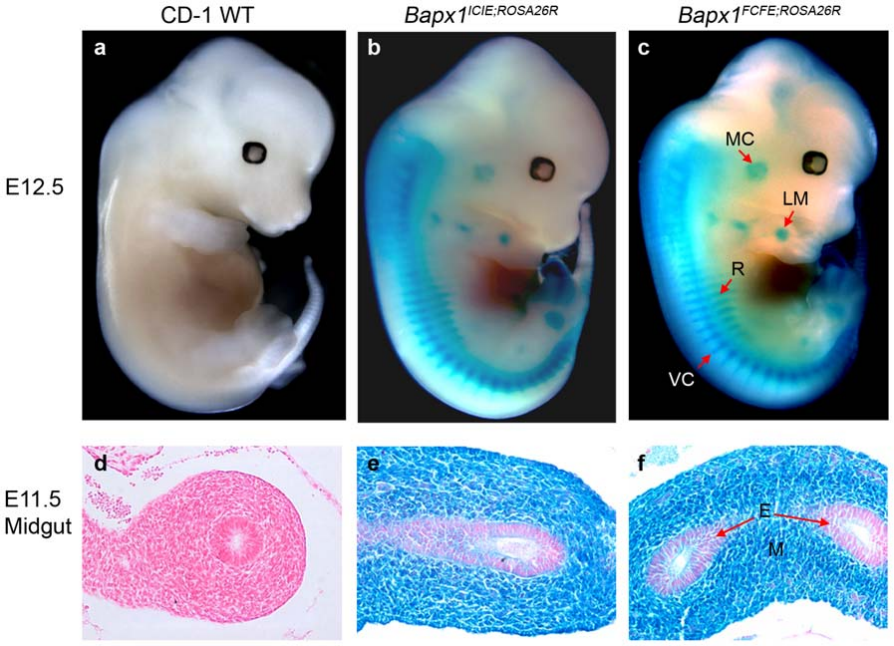
Functional analysis of Cre activity in the *Bapx1^ICIE;Rosa26R^* and *Bapx1^FCFE;Rosa26R^* mice. (a–c) Whole-mount E12.5 *Bapx1^ICIE;Rosa26R^* and *Bapx1^FCFE;Rosa26R^* embryos stained with X-gal showing β-galactosidase activity in the correct anatomical context of *Bapx1*. (d–f) Transverse sections of E11.5 embryos confirmed X-gal stained cells in the mesenchymal cells of the midgut and not in the endothelium. MC, Meckel's cartilage; LM, limb mesenchyme; VC, vertebral column; R, ribs; M, mesenchyme; E, endothelium.

**Figure 5:**
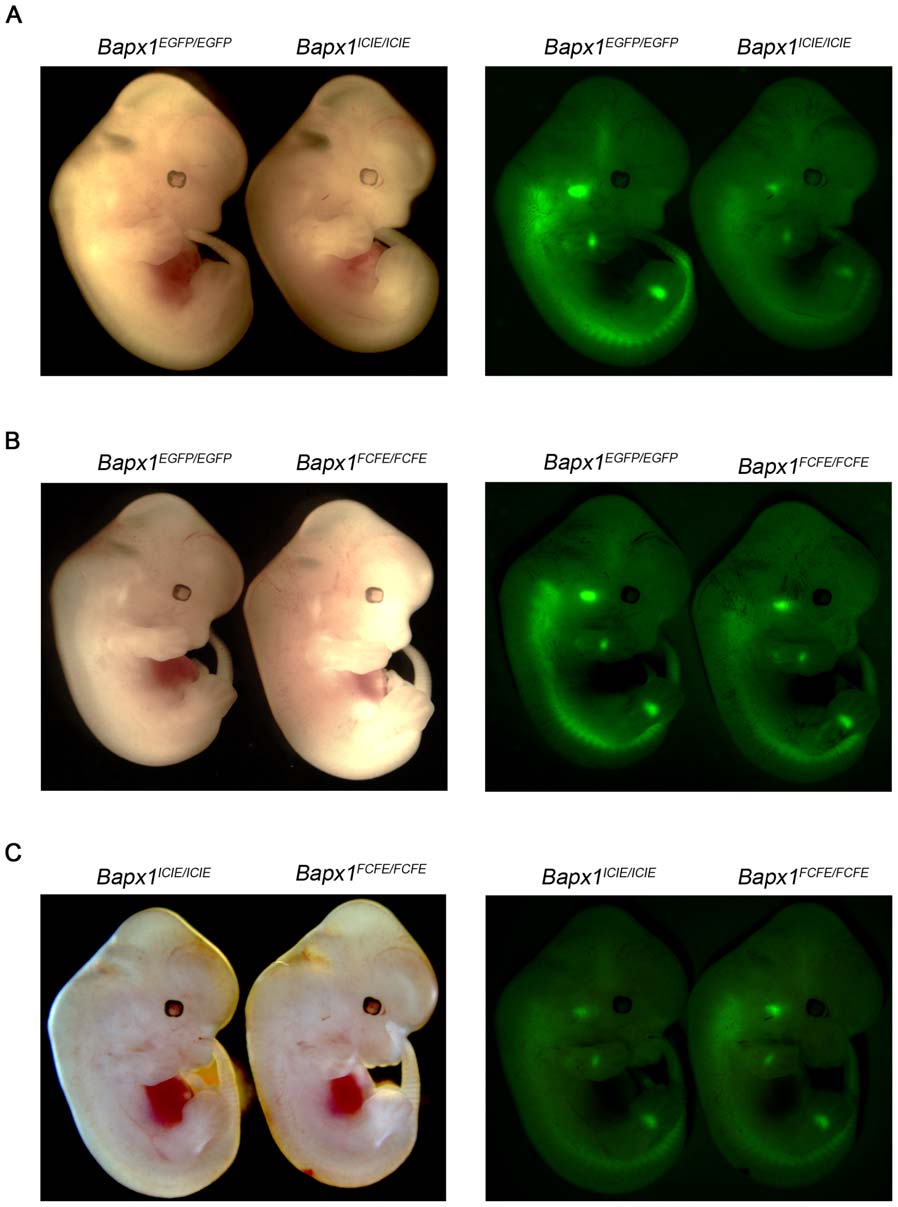
EGFP expression mediated by IRES and F2A at the *Bapx1* locus. Homozygous E12.5 *Bapx1^EGFP/EGFP^*, *Bapx1^ICIE/ICIE^ and Bapx1^FCFE/FCFE^* embryos imaged under white light (left panel) and fluorescence microscope (right panel). EGFP expression recapitulates Bapx1 expression domains in all three *Bapx1*-targeted mouse lines. (a) In *Bapx1^EGFP/EGFP^* vs. *Bapx1^ICIE/ICIE^*, the former clearly showed brighter fluorescence. (b) In *Bapx1^EGFP/EGFP^* vs. *Bapx1^FCFE/FCFE^*, both had comparable fluorescence intensities. (c) In *Bapx1^ICIE/ICIE^ vs. Bapx1^FCFE/FCFE^*, the latter displayed a slightly higher EGFP fluorescence.

**Figure 6:**
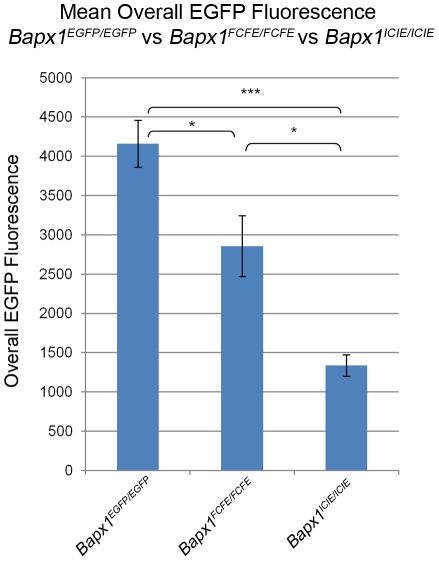
Mean overall EGFP fluorescence of E12.5 *Bapx1^EGFP/EGFP^*, *Bapx1^FCFE/FCFE^* and *Bapx1^ICIE/ICIE^* embryos. Mean overall EGFP fluorescence of E12.5 *Bapx1^EGFP/EGFP^* (4159.56±297.81; n = 7), *Bapx1^FCFE/FCFE^* (2856.74±387.58; n = 11) and *Bapx1^ICIE/ICIE^* (1338.13±137.83; n = 9) embryos analyzed by flow cytometry. Overall EGFP fluorescence was calculated by multiplying MFI and percentage of EGFP-positive cells. Two-tailed Student's T test, *p<0.05, ***p<0.0001.

**Figure 7:**
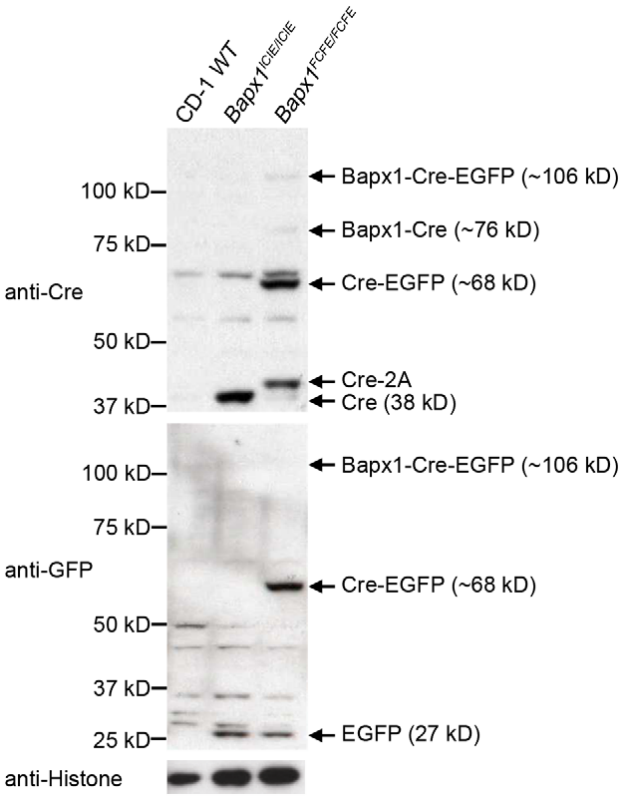
Efficiency of the IRES and the F2A at the *Bapx1* locus assessed by Western blotting. Lysates from E12.5 CD-1 WT, *Bapx1^ICIE/ICIE^* and *Bapx1^FCFE/FCFE^* embryos were resolved on SDS-PAGE and immunoblotted with anti-Cre (top panel) and anti-GFP (middle panel) antibodies. Top panel: Faint bands were detected for Bapx1-Cre-EGFP and Bapx1-Cre fusion proteins at the predicted molecular weights in *Bapx1^FCFE/FCFE^*; significant amounts of Cre-EGFP fusion proteins and Cre-2A (2A – residual 23 amino acids of F2A) were also detected. No fusion proteins or Cre-2A, but Cre (38 kD), were detected in *Bapx1^ICIE/ICIE^*. Middle panel: Cre-EGFP but not Bapx1-Cre-EGFP fusion protein was detected in *Bapx1^FCFE/FCFE^*; EGFP of predicted molecular weight was detected in both *Bapx1^ICIE/ICIE^* and *Bapx1^FCFE/FCFE^*, but not in the negative control (CD-1 WT). Bottom panel: immunoblotting with anti-histone antibody showed equal loading.

To further explore if the ‘skipping’ efficiency was dependent on the upstream gene or the downstream *EGFP* reporter, the *Cre recombinase* gene was appended in the second position, between *Bapx1* and *EGFP*, to generate a tricistronic knock-in mouse line (*Bapx1^FCFE^*) (Fig. 1B). We also generated a *Bapx1^ICIE^* mouse line in parallel to complete our *in vivo* evaluation of multicistronic co-expression efficacy and efficiency of the IRES and F2A sequences (Fig. 1B). The activity and expression of each of the proteins (Cre, EGFP and Bapx1) were analyzed by X-gal staining, fluorescence microscopy, flow cytometry and WB of developing mouse embryos (Figs. 4, 5, 6 and 7).

### Activity and spatio-temporal expression of *Cre* assessed by X-gal staining

To assess the recombinase activity of Cre, the *ROSA26* conditional reporter mice (*ROSA26R*, stock number 003309, JAX®) were first intercrossed with *Bapx1^ICIE^* and *Bapx1^FCFE^* mice to generate embryos heterozygous for both alleles. The floxed translation-stop sequence in the *Rosa26R* allele recombines in the presence of Cre recombinase and *lacZ* gene expression is subsequently activated and restricted to the Cre-expressing cells and their progeny [30].

In our study, Cre-mediated recombination of the *ROSA26R* allele in both the *Bapx1^ICIE;Rosa26R^* and *Bapx1^FCFE;Rosa26R^* mice was apparent at E9.5 in the cephalic mesenchyme, somites and splanchnic mesoderm [31]. At E12.5, strong X-gal staining was observed in the expected regions of the cartilage anlagen of the axial and appendicular skeleton as well as the gut mesoderm of both the *IRES-Cre* and *F2A-Cre* mice (Fig. 4a–c). Transverse sections of E11.5 embryos displaying X-gal staining that was confined to the mesenchyme of the midgut further demonstrated the tissue-specificity of the Cre recombinase activity (Fig. 4d–f). In summary, the Cre in both the IRES and F2A mice was active and functional in the known Bapx1 expression domains as revealed by the X-gal staining.

### Functional analysis of EGFP by fluorescence intensity and flow cytometry

E12.5 embryos from *Bapx1^ICIE/ICIE^* and *Bapx1^FCFE/FCFE^* mouse lines were harvested on the same day and green fluorescence of homozygous embryos was detected by fluorescence microscopy. Fluorescence in the *Bapx1^ICIE/ICIE^* embryos was observed to be slightly weaker than that in the *Bapx1^FCFE/FCFE^* embryos (Fig. 5C). The mean overall EGFP fluorescence level for the *Bapx1^FCFE/FCFE^* embryos (2856.74±387.58; n = 11) was 2.13-fold higher (p<0.05) than that in the *Bapx1^ICIE/ICIE^* embryos (1338.13±137.83; n = 9) (Fig. 6 and Table 2 and S2).

To determine which of the embryos recapitulate the endogenous *Bapx1* expression level, we analyzed another construct, *Bapx1^EGFP^*, where *EGFP* was inserted right after the translational start site so that it was under the direct control of the *Bapx1* promoter without being linked to any 2A or IRES sequence (Fig. 1B). Based on our expression analysis and chromatin immunoprecipitation (ChIP)-Seq analysis (data not shown), we could exclude a negative feedback autoregulation for *Bapx1* as there was no up-regulation of *Bapx1* transcripts in the *Bapx1^null^* embryos. Therefore, the EGFP protein expression of the *Bapx1^EGFP/EGFP^* embryos should represent the endogenous levels of Bapx1 protein. For this reason, the overall EGFP fluorescence levels of the *Bapx1^ICIE/ICIE^* and *Bapx1^FCFE/FCFE^* embryos were compared with that of the *Bapx1^EGFP/EGFP^* embryos. A much higher level of green fluorescence was seen in the E12.5 *Bapx1^EGFP/EGFP^* embryos in contrast to the *Bapx1^ICIE/ICIE^* embryos of the same stage (Fig. 5A). Flow cytometry analysis also showed a 3.11-fold greater (p<0.0001) mean overall EGFP fluorescence levels of the *Bapx1^EGFP/EGFP^* embryos (4159.56±297.81; n = 7) compared to that of the *Bapx1^ICIE/ICIE^* embryos (Fig. 6 and Table 2 and S2). Likewise, a comparison of the *Bapx1^EGFP/EGFP^* embryos with the *Bapx1^FCFE/FCFE^* embryos revealed a higher mean overall EGFP fluorescence level in the former by 1.46-fold (p<0.05) evident from fluorescence microscopy and FACS analysis (Figs. 5B, 6, Table 2 and S2). Nevertheless, this deviation from the endogenous Bapx1 protein expression level was 36.5% less than that of the *Bapx1^ICIE/ICIE^* embryos (Table S2 legend). Thus, the EGFP protein expression in the *Bapx1^FCFE/FCFE^* embryos more closely resembled the endogenous Bapx1 protein expression level than that of the *Bapx1^ICIE/ICIE^* embryos.

### Co-translational 2A ‘skipping’ efficiency in the *Bapx1^FCFE^* mice

We analyzed Bapx1, Cre recombinase and EGFP protein expression by Western blotting using cell lysates from enriched tissues (limbs and vertebral column) of homozygous E12.5 *Bapx1^ICIE/ICIE^* and *Bapx1^FCFE/FCFE^* embryos. Protein extract from wild-type (WT) CD-1 mouse embryos was included as a negative control for Cre recombinase and EGFP proteins. Migrations of Cre (38 kD) and EGFP (27 kD) proteins from the *Bapx1^ICIE/ICIE^* embryos on the SDS-PAGE gel were consistent with their predicted molecular weights as detected by anti-Cre and anti-GFP antibodies (Fig. 7).

We then examined the efficiency of co-translational 2A ‘skipping’ in the *Bapx1^FCFE/FCFE^* mice. Interestingly, the ‘skipping’ at the F2A between *Bapx1* and *Cre* was found to be >99% efficient as only an insignificant amount of Bapx1-Cre and Bapx1-Cre-EGFP fusion proteins were detected by the anti-Cre antibody (Fig. 7 top panel). Then again, the ‘skipping’ at the second F2A, between *Cre* and *EGFP*, was highly inefficient yielding 55.3% and 62.2% of Cre-EGFP fusion protein as identified by both the anti-Cre and anti-GFP antibodies respectively. Bapx1-Cre-EGFP, however, was not detected by the anti-GFP antibody which, as mentioned earlier, could be attributed to its lower sensitivity (Fig. 7 and Table S3).

Lastly, similar to the observations for Sox9 (Sox9-2A), we also detected a slightly higher molecular weight Cre protein (Cre-2A) in the *Bapx1^FCFE/FCFE^* lysate, indicating that the RAKR protease motif upstream of the second F2A, failed to induce complete cleavage of the residual F2A peptide at the C-terminus of Cre (Fig. 7). As the available anti-Bapx1 antibody was not sensitive enough to detect Bapx1 from unpurified embryo lysates, we could not evaluate if the RAKR preceding the first F2A was functional (data not shown).

## Discussion

The development of technologies such as FACS to isolate cells, and expression profiling, RNA-Seq and ChIP-Seq for genome-wide analyses of gene functions have brought about an immense need to co-express genes with reporter proteins for cell tracking and high-purity enrichment. Furthermore, the employment of Cre recombinase to achieve conditional gene ablation has become a prevalent method to study genes that cause prenatal lethality [32], [33], [34]. Using the IRES and/or the 2A peptide to generate such multicistronic vectors has become the norm to tag endogenous genes with reporter or recombinase proteins [5], [17], [33], [35], [36]. Hence, we have adopted these two strategies for our study to assess their applicability in an *in vivo* scenario, comparing the IRES and the F2A peptide for the first time in stable mouse lines showing locus-specific expression of the transgenes.

It is appropriate to study TFs as a proof-of-principle as there is a widespread interest in TFs owing to their key roles as regulatory molecules and also their importance in reprogramming of somatic cells to iPS cells [7]. *Sox9*, in particular, was of interest as its functions are well-studied and it is expressed at an early embryonic stage which made it technically feasible for experimentation [26]. Similarly, *Bapx1* was chosen as the alternative locus of comparison owing to its similar tissue expression domains as *Sox9* and its appearance early in development [28].

From the EGFP fluorescence levels seen in the fluorescence microscopy and flow cytometry of the *Sox9*- and *Bapx1*-targeted embryos, we observed that the F2A-mediated multicistronic constructs consistently produced higher EGFP levels than the corresponding IRES-constructs. Furthermore, when compared with the *Bapx1^EGFP/EGFP^* embryos, the *Bapx1^FCFE/FCFE^* embryos had a near stoichiometric protein expression of EGFP and Bapx1. Conversely, *Bapx1^ICIE/ICIE^* showed a greater deviation from the endogenous level of *Bapx1* expression. These results conform to the known properties of the IRES producing lower amounts of the downstream protein in relation to the upstream protein. Nevertheless, both the F2A and the IRES co-expressed functional proteins at acceptable levels in the *Sox9* and the *Bapx1* loci.

Contrary to the conclusion of Lorens *et al* 2004 [37] who attributed the complete absence of fusion protein to the inclusion of the GSG linker, we observed a substantial amount of fusion protein despite its addition preceding the F2A sequence. In fact, other studies [16], [38] that used the GSG linker sequence still showed an inefficient ‘skipping’ which is consistent with our results. In addition, the RAKR motif used to remove the residual 2A peptide from the upstream protein also proved ineffective in our study, as shown by the presence of Sox9-2A and Cre-2A proteins. This result clearly differs from what was reported by Fang *et al* 2005 [2]. To our knowledge no one has used both the GSG linker and the RAKR motif in conjunction. Hence, we believe unknown effects of this partnership might have led to the dysfunction of the furin protease site. This also proves that such linker sequences and the furin motif are not fool-proof solutions for inefficient ‘skipping’ and the removal of residual peptides respectively.

The presence of 42.2% Sox9-EGFP fusion protein led us to investigate further if the upstream protein in context might hinder the ‘skipping’ mechanism of the F2A peptide. Therefore, we analyzed the F2A ‘skipping’ efficiency in a construct with a different upstream protein (Bapx1).

To avoid confounding our results with potential stage-specific alterations to the ‘skipping’ function, we kept the developmental stage of interest (E12.5) constant in our study. As Bapx1 is a moderately expressed protein and the anti-Bapx1 antibody was not adequately sensitive, we only managed to detect it from the *FLAG_3_-Bapx1^FE^* embryo lysates after purification. Surprisingly, despite changing the upstream protein in context (*FLAG_3_-Bapx1^FE^*), we still observed 49.4% FLAG_3_-Bapx1-EGFP fusion protein thus validating our conclusions drawn from the *Sox9^FE^* construct. Hence, we can be more confident that neither the upstream protein nor the locus was responsible for the ‘skipping’ inefficiency of the F2A. This was further corroborated by the results from the *Bapx1^FCFE/FCFE^*, whereby Bapx1 was shown not to affect the ‘skipping’ process in the first F2A due to the negligible amounts of Bapx1-Cre fusion protein detected. This also implied that Cre does not have an adverse effect on the 2A-mechanism. However, even while maintaining the locus but changing the gene upstream of *EGFP*, from *Bapx1* to *Cre* in *Bapx1^FCFE/FCFE^*, we still saw 55.3% and 62.2% fusion protein (Cre-EGFP) relative to Cre and EGFP proteins respectively. This indicated that the ‘skipping’ at the second F2A was highly inefficient. Since EGFP was the common protein in all the constructs, we surmised that EGFP might be the causal agent behind the inefficient ‘skipping’ mechanism.

Upon scrutiny of prior publications which also used the F2A in their constructs, fusion proteins were observed in all instances where EGFP was the concatenated protein, in both *in vitro* translation or *in vivo* cell culture and animal models [6], [16], [17], [39]. Besides, in Furler *et al* 2001 [17] and Kim *et al* 2011 [16], a significant degree of ‘skipping’ inefficiency was observed even when *EGFP* was upstream of the F2A sequence, proving that what was observed in our study was not a positional effect [40]. This strongly supports our hypothesis that EGFP may have an inhibitory effect on the F2A-mediated ‘skipping’ mechanism, the basis of which is yet unclear. In their recent publication, Felipe *et al* 2010 [19] proposed that the inhibition of the 2A process might be dependent on the upstream nascent protein. On the other hand, based on our results, it is evident that there is much more to the mechanism of the 2A-mediated ‘skipping’ which remains obscure to date. Structural investigations and partial deletion experiments of *EGFP* in bicistronic constructs, similar to what Felipe *et al* 2010 [19] had performed in their investigation, may help to reveal the mechanism.

In our study, we have clearly demonstrated that the F2A peptide and the IRES elements are functional in multicistronic constructs when expressed in a stable mouse line. The F2A maintains a more reliable expression level of the appended downstream cistron than the IRES. Additionally, our vital finding that the predominantly-used EGFP reporter likely hinders the ‘skipping’ mechanism of the F2A peptide serves as a caution to researchers who utilize it in their F2A-based multicistronic constructs. Ultimately, the 2A peptide remains unchallenged as the ideal strategy for co-expressing multiple proteins *in vivo* and researchers often opt for EGFP as the reporter in mouse models because it is highly photostable and one of the brightest fluorescent proteins [20]. The presence of a fusion protein from inefficient ribosomal ‘skipping’ has often been ignored, but it is imperative that we do not overlook the implications of such a fusion protein in an *in vivo* system. As there are increasing numbers of studies employing expression profiling and next-generation sequencing to elucidate gene regulation at cellular resolution, it is important to note that even minute amounts of fusion protein may alter the genomic architecture and lead to a false representation of transcripts and binding sites. Therefore, we ought to employ strategies that will give us equivalent expression levels on top of producing discrete translational products. Hence, to alleviate some of the issues, other 2A peptides (E2A, P2A, T2A) could be verified for their compatibility with EGFP. Furthermore, other reporter proteins could be explored for use in the F2A-based constructs. While several others propose using GSG linkers and longer versions of the F2A to counter the ‘skipping’ problem [15], [19], [37], we believe these are not fail-safe solutions, and should be assessed carefully for every protein in context.

## Materials and Methods

### Ethics Statement

All animal procedures were performed according to the Singapore A*STAR Biopolis Biological Resource Center (BRC) Institutional Animal Care and Use Committee (IACUC) guidelines and the IACUC protocols employed were reviewed and approved by the aforementioned committee before any animal procedures were undertaken for the study described here (IACUC Protocol No: 080348 and 080377).

### Bacterial Artificial Chromosome (BAC) modification and *Bapx1* targeting

Murine BAC clone RP24-148P5 derived from the C57BL/6J mouse strain, containing over 160 kb of genomic DNA flanking the *Bapx1* gene was obtained from the BACPAC Resources Centre at Children's Hospital Oakland Research Institute (CHORI). The BAC was modified by homologous recombination using the Quick and Easy BAC modification kit (Gene Bridges) following the manufacturer's instruction. The *IRES-Cre-IRES-EGFP-FRT-PGK-gb2-Neo-FRT* cassette was inserted into the 3′UTR of *Bapx1*, whereas the *F2A-Cre-F2A-EGFP-FRT-PGK-gb2-Neo-FRT* cassette was inserted immediately before the translational stop codon of *Bapx1* (Fig. 1B). A 20.6 kb fragment (IRES clone) and a 19.6 kb fragment (F2A clone) spanning the whole coding region of *Bapx1* were then subcloned into a minimal vector using the BAC subcloning kit (Gene Bridges). The resulting targeting vectors were linearized and electroporated into V6.4 mouse embryonic stem cells (ESC) [41], to generate *Bapx1^ICIE^* and *Bapx1^FCFE^* ESC clones. The gene targeting events were confirmed by Southern blotting and the correctly targeted ESC clones with a normal karyotype were subsequently microinjected into 2–8 cell stage embryos isolated from C57BL/6J mice to generate germ-line transmitting chimeric mice [42]. Subsequent heterozygous progeny was bred to the *ROSA-Flpe* mice (Stock number 3946, JAX®) for the excision of the *FRT*-flanked *neomycin* cassette in the targeted *Bapx1* allele. Details of the construct design for *Bapx1^ICIE^* and *Bapx1^FCFE^* have been described in [31] while those for *Sox9^IE^*, *Sox9^FE^*, *FLAG_3_-Bapx1^FE^* and *Bapx1^EGFP^* will be described in another manuscript that is in preparation.

### Southern blotting and PCR genotyping

Genomic DNA was extracted from ESC cells or tail biopsies using phenol-chloroform following overnight Proteinase K digestion (Sigma, final concentration of 0.1 mg/ml and 0.5 mg/ml for cells and tails respectively) at 55°C. DNA (10 ug) digested with the appropriate restriction enzymes was separated on 0.8% agarose gels, denatured with 0.5 M NaOH and transferred to positively-charged nylon membranes (Amersham Hybond-N+). Membranes were hybridized overnight with the appropriate Digoxigenin (DIG)-labeled probes (Roche PCR DIG Probe Synthesis Kit) at 42°C following a 4-hour (h) prehybridization (Roche DIG Easy Hyb). The following day, membranes were washed [2× SSC for 10 minutes (mins) at room temperature (RT) then 0.5× SSC, 0.1% SDS for 2×15 mins at 60°C], blocked (1× Roche Blocking Reagent) and probed with anti-DIG-AP antibody (Roche) at 1∶5000 dilution in 1× blocking buffer, each step for 30 mins at RT. Membranes were washed again in 1× MABT, 2×15 mins at RT, rinsed briefly in detection buffer and incubated with CSPD at 37°C for 10 mins before visualization on X-ray films. Routine mouse genotyping was performed by PCR with the following primer pairs and amplicon: *EGFP* (5′ CCTACGGCGTGCAGTGCTTCAGC 3′ and 5′ CGGCGAGCTGCACGCTGCCGTCC 3′; 345 bp); primer pair 1 (5′ GACCCCAGGAGCTCTGCAC 3′ and 5′ GGGCTGAAAGGATTCTGCAC 3′; *WT* allele: 293 bp, *Bapx1^ICIE^* allele: 3.4 kb) and primer pair 2 (5′ AAGAAAGTGGCCGTAAAGGTG 3′ and 5′ ACCCTGGATAGGTGTGTCAAGT 3′; *WT* allele: 320 bp, *Bapx1^FCFE^* allele: 2.7 kb).

### Flow cytometric analysis

Mouse embryos were dissociated into single cells by repeated pipetting in an enzymatic buffer comprising Collagenase I & II (100 U/ml; Gibco), DNAse (50 U/ml; Sigma) and 0.05% Trypsin (Gibco). The cells were filtered through a 100 uM followed by a 40 uM cell strainer before centrifugation at 2000 rpm for 5 mins. The cell pellet obtained was resuspended in 5% fetal bovine serum, 4 mM EDTA in Leibovitz's L-15 medium for flow cytometry analysis. E12.5 CD-1 WT embryos were used for gating. Overall EGFP fluorescence (mean fluorescence intensity (MFI) multiplied by the percentage of EGFP-positive cells) was measured with the FACSAria flow cytometer (BD Biosciences) and analyzed using the FACSDiva Version 6.1.3 software.

### Statistical Evaluation

For all overall EGFP fluorescence values (MFI×percentage of EGFP-positive cells), the mean and standard errors were calculated using the data analysis tool (Analysis ToolPak) in Microsoft Excel X. Student's T-tests (two-tailed) with 95% confidence intervals were performed for all pair-wise comparisons to analyze the differences in the overall EGFP fluorescence levels.

### Mouse crosses

The Cre-Tester line *B6;129^S4-Gt(ROSA)26Sortm1Sor/J(ROSA26R)^* was purchased as frozen embryos from JAX® (Stock number 003309. Abbreviated as *Rosa26R*) and re-derived by the Biological Resource Centre (A*STAR). Cre-tester mice were mated to the created mouse lines, *Bapx1^ICIE^* and *Bapx1^FCFE^* for the generation of double heterozygous embryos, *Bapx1^ICIE;Rosa26R^* and *Bapx1^FCFE;Rosa26R^*. E0.5 was defined as the day the vaginal plug was detected. Upon Cre recombination in Bapx1-expressing cells, *lacZ* gene expression was activated and reflected by β-galactosidase activity in these tissues.

### Whole-mount X-gal staining of E11.5 and E12.5 mouse embryos

Mouse embryos were harvested in cold Leibovitz medium and critically staged using morphological criteria established by M.H. Kaufmann. These embryos were fixed for 5 mins (<E12.5) or 15 mins (>E12.5) in 4% paraformaldehyde (PFA) on ice, followed by 3×5 mins washes in ice-cold PBS. Embryos were left for colour development in X-gal staining solution (2 mM MgCl_2_; 0.01% deoxycholic acid; 0.02% Igepal CA-630; 0.1% (1 mg/ml) X-Gal in DMF; 5 mM K4; 5 mM K3 in 1×PBS, pH 8) at 4°C in the dark for 48 h with agitation. Stained embryos were subsequently rinsed and post-fixed.

### Paraffin embedding and sectioning

X-gal-stained mouse embryos were post-fixed in 4% PFA at 4°C overnight, washed with PBS, dehydrated through 50% and 70% ethanol/PBS for 15 mins each before being placed in an automated tissue processor (Leica TP 1020) for further dehydration through 70%, 90%, 95% ethanol/PBS and 100% ethanol, followed by Histo-clear® (substituted for xylene) and paraffin embedding. Paraffin-embedded embryos were sectioned at 10 microns with a Leica RM 2165 microtome. Sections were placed on polysine-coated glass slides (Fischer-Scientific) and subsequently mounted with glycerol gelatin (Sigma). All sections were photographed using Zeiss Axio Imager Z1 microscope.

### Western blotting

Whole or dissected mouse embryos were homogenized and protein lysates were obtained following instructions in the NE-PER® Nuclear and Cytoplasmic Extraction Reagent kit (Thermo Scientific). Bradford protein assay was used to measure the protein concentrations (BioRad Cat. # 500-0205). Equal amounts of cell lysates were loaded for each lane. Proteins were separated in NuPAGE® 4%–12% Bis-Tris gels (Invitrogen) and transferred to a PVDF membrane (Biorad Immun-Blot™) at 25 volts for 30 mins with Biorad Trans-Blot® Semi-Dry Transfer Cell. Membranes were blocked in 5% skimmed milk (BD Difco) in TBST for 1 h at RT, incubated with goat anti-Sox9 (AF3075 (R&D Systems); 1∶1000 dilution), rabbit anti-Bapx1 (ab56029 (Abcam); 1∶2000 dilution), rabbit anti-GFP (sc-8334 (Santa Cruz); 1∶ 200 dilution), rabbit anti-Cre (pRB-106C (Covance); 1∶500 dilution) or rabbit anti-Histone (ab1791 (Abcam); 1∶1000 dilution) in 3% BSA in TBST for 1 h at RT and washed in TBST for 4×15 mins before incubation with HRP-conjugated donkey anti-rabbit antibody (NA934 (GE healthcare); 1∶ 10,000) or HRP-conjugated donkey anti-goat antibody (sc2020 (Santa Cruz); 1∶5000) in 3% BSA in TBST for 1 h at RT. Membranes were then washed in TBST for 3×10 mins at RT. Relevant proteins were detected with Supersignal™ West Pico enhanced chemiluminescence (ECL) reagents (Thermo Scientific) and exposed onto X-ray films. Densitometric analysis of the protein bands on the X-ray films were performed by measuring protein band densities (OD/mm^2^) using the GS-800 Calibrated Densitometer and analyzing the results using the Quantity One 4.5.2 software. All values were normalized to their respective histone densities.

## Supporting Information

Table S1 **MFI, percentage of EGFP^+^ cells and overall EGFP fluorescence of E12.5** ***Sox9*** **mouse embryos.** Raw values of MFI and percentage of EGFP^+^ for each *Sox9^IE/IE^* and *Sox9^FE/FE^* mouse embryo. Overall EGFP fluorescence was calculated by multiplying MFI and percentage of EGFP^+^ cells. MFI – Mean fluorescence intensity; EGFP – Enhanced green fluorescence protein; SE –Standard error.
(DOC)

Table S2 **MFI, percentage of EGFP^+^ cells and overall EGFP fluorescence of E12.5** ***Bapx1*** **mouse embryos.** Raw values of MFI and percentage of EGFP^+^ for each *Bapx1^EGFP/EGFP^*, *Bapx1^ICIE/ICIE^* and *Bapx1^FCFE/FCFE^* mouse embryo. Overall EGFP fluorescence was calculated by multiplying MFI and percentage of EGFP^+^ cells. Percentage difference in deviation of mean overall fluorescence from endogenous levels between F2A and IRES *Bapx1* embryos = [(A–B)−(A–C)]/A *100% = 36.5%. MFI – Mean fluorescence intensity; EGFP – Enhanced green fluorescence protein; SE –Standard error.
(DOC)

Table S3 **Densitometry analysis of the Western blots.** Raw densitometry values calculated for the protein bands in the Western blots of figures 2D, 3 and 7. 2A - residual 23 amino acids of F2A; EGFP – Enhanced green fluorescence protein; OD – optical density.
(DOC)

## References

[1] Mir F, Kaufmann SH, Eddine AN (2009). A multicistronic DNA vaccine induces significant protection against tuberculosis in mice and offers flexibility in the expressed antigen repertoire.. Clin Vacc Immunol.

[2] Fang J, Qian J, Yi S, Harding TC, Tu G (2005). Stable antibody expression at therapeutic levels using the 2A peptide.. Nat Biotechnol.

[3] Szymczak A, Workman CJ, Wang Y, Vignali KM, Dilioglou S (2004). Correction of multi-gene deficiency in vivo using a single ‘self-cleaving’ 2A peptide-based retroviral vector.. Nat Biotechnol.

[4] Yang S, Cohen CJ, Peng PD, Zhao Y, Cassard L (2008). Development of optimal bicistronic lentiviral vectors facilitates high-level TCR gene expression and robust tumor cell recognition.. Gene Therapy.

[5] Hsiao E, Yoshinaga Y, Nguyen TD, Musone SL, Kim JE (2008). Marking embryonic stem cells with a 2A self-cleaving peptide: A NKX2-5 emerald GFP BAC reporter.. Plosone.

[6] Ibrahimi A, Velde GV, Reumers V, Toelen J, Thiry I (2009). Highly efficient multicistronic lentiviral vectors with peptide 2A sequences.. Human Gene Ther.

[7] Carey B, Markoulaki S, Hanna J, Saha K, Gao Q (2009). Reprogramming of murine and human somatic cells using a single polycistronic vector.. Proc Natl Acad Sci U S A.

[8] De Felipe P (2002). Polycistronic viral vectors.. Curr Gene Ther.

[9] Jang S, Krausslich HG, Nicklin MJH, Duke GM, Palmenberg AC (1988). A segment of the 5′ nontranslated region of encephalomyocarditis virus RNA directs internal entry of ribosomes during in vitro translation.. J Virol.

[10] Hellen C, Sarnow P (2001). Internal ribosome entry sites in eurkaryotic mRNA molecules.. Genes Dev.

[11] Vagner S, Galy B, Pyronnet S (2001). Irresistible IRES.. EMBO Report.

[12] Licursi M, Chrisitan SL, Pongnonpparat T, Hirasawa K (2011). In vitro and in vivo comparison of viral and cellular internal ribosome entry sites for bicistronic vector expression.. Gene therapy.

[13] Donnelly M, Gani D, Flint M, Monaghan S, Ryan MD (1997). The cleavage activities of aphthovirus and cardiovirus 2A proteins.. J Gen Virol.

[14] De Felipe P (2004). Skipping the co-expression problem: the new 2A “CHYSEL” technology.. Genetic Vaccines and Therapy.

[15] Donnelly M, Hughes LE, Luke G, Mendoza H, Dam E (2001). The ‘cleavage’ activities of foot-and-mouth disease virus 2A site-directed mutants and naturally occurring ‘2A-like’ sequences.. J Gen Virol.

[16] Kim J, Lee SR, Li LH, Park HJ, Park JH (2011). High cleavage efficiency of a 2A peptide derived from porcine teschovirus-1 in human cell lines, zebrafish and mice.. Plosone.

[17] Furler S, Paterna JC, Weibel M, Bueler H (2001). Recombinant AAV vectors containing the foot and mouth disease virus 2A sequence confer efficient bicistronic gene expression in cultured cells and rat substantia nigra neurons.. Gene Therapy.

[18] Hasegawa K, Cowan AB, Nakatsuji N, Suemori H (2007). Efficient multicistronic expression of a transgene in human embryonic stem cells.. Stem Cell.

[19] De Felipe P, Luke GA, Brown JD, Ryan MD (2010). Inhibition of 2A mediated ‘cleavage’ of certain artificial polyproteins bearing N-terminal signal sequences.. Biotechnol J.

[20] Day R, Davidson MW (2009). The fluorescent protein palette: tools for cellular imaging.. Chem Soc Rev.

[21] Nakamura Y, Yamamoto K, He X, Otsuki B, Kim Y (2011). Wwp2 is essential for palatogenesis mediated by the interaction between Sox9 and mediator subunit 25.. Nat Commun.

[22] McLenachan S, Goldshmit Y, Fowler KJ, Voullaire L, Holloway TP (2008). Transgenic mice expressing the Peripherin-EGFP genomic reporter display intrinsic peripheral nervous system fluorescence.. Transgenic Res.

[23] Akiyama H, Chaboissier MC, Martin JF, Schedl A, de Crombrugghe B (2002). The transcription factor Sox9 has essential roles in successive steps of the chondrocyte differentiation pathway and is required for expression of Sox5 and Sox6.. Genes Dev.

[24] Akiyama H (2008). Control of chondrogenesis by the transcription factor Sox9.. Modern Rheumatology.

[25] Bi W, Deng JM, Zhang Z, Behringer RR, de Crombrugghe B (1999). Sox9 is required for cartilage formation.. Nat Genet.

[26] Zhao Q, Eberspaecher H, Lefebvre V, de Crombrugghe B (1997). Parallel expression of Sox9 and Col2a1 in cells undergoing chondrogenesis.. Dev Dyn.

[27] Holst J, Vignali KM, Burton AR, Vignali DA (2006). Rapid analysis of T-cell selection in vivo using T cell-receptor retrogenic mice.. Nat Method.

[28] Tribioli C, Frasch M, Lufkin T (1997). Bapx1: an evolutionary conserved homologue of the Drosophila bagpipe homeobox gene is expressed in splanchnic mesoderm and the embryonic skeleton.. Mech Dev.

[29] Tribioli C, Lufkin T (1999). The murine Bapx1 homeobox gene plays a critical role in embryonic development of the axial skeleton and spleen.. Development.

[30] Soriano P (1999). Generalized lacZ expression with the ROSA26 Cre reporter strain.. Nat Gen.

[31] Sivakamasundari V, Chan HY, Yap SP, Xing X, Kraus P (2011). New Bapx1(Cre-EGFP) mouse lines for lineage tracing and conditional knockout studies.. Genesis.

[32] Howard P, Howard TL, Maurer RA (2010). Generation of mice with a conditional allele for Ift172.. Transgenic Res.

[33] Nakamura Y, Yamamoto K, He X, Otsuki B, Kim Y (2011). Wwp2 is essential for palatogenesis mediated by the interaction between Sox9 and mediator subunit 25.. Nat Commun.

[34] Yap S, Xing X, Kraus P, Sivakamasundari V, Chan HY (2011). Generation of mice with a novel conditional null allele of the *Sox9* gene.. Biotech Lett.

[35] Ghim C, Lee SK, Takayama S, Mitchell RJ (2010). The art of reporter proteins in science: past, present and future applications.. BMB Reports.

[36] Provost E, Rhee J, Leach SD (2007). Viral 2A peptides allow expression of multiple proteins from a single ORF in transgenic zebrafish embryos.. Genesis.

[37] Lorens J, Pearsall DM, Swift SE, Peelle B, Armstrong R (2004). Stable, stoichiometric delivery of diverse protein functions.. J Biochem Biophys Method.

[38] Funston G, Kallioinen SE, De Felipe P, Ryan MD, Iggo RD (2008). Expression of heterologous genes in oncolytic adenoviruses using picornaviral 2A sequences that trigger ribosome skipping.. J Gen Virol.

[39] Chinnasamy D, Milsom MD, Shaffer J, Neuenfeldt J, Shaaban AF (2006). Multicistronic lentiviral vectors containing the FMDV 2A cleavage factor demonstrate robust expression of encoded genes at limiting MOI.. J Virol.

[40] Rothwell D, Crossley R, Bridgeman JS, Sheard V, Zhang Y (2010). Functional expression of secreted proteins from a bicistronic retroviral cassette based on FMDV 2A can be position-dependent.. Human Gene Ther.

[41] Eggan K, Akutsu H, Loring J, Jackson-Grusby L, Klemm M (2001). Hybrid vigor, fetal overgrowth, and viability of mice derived by nuclear cloning and tetraploid embryo complementation.. Proc Natl Acad Sci U S A.

[42] Kraus P, Leong G, Tan V, Xing X, Goh JW (2010). A more cost effective and rapid high percentage germ-line transmitting chimeric mouse generation procedure via microinjection of 2-cell, 4-cell, and 8-cell embryos with ES and iPS cells.. Genesis.

