# Supplementary material for: Comparison of IRES and F2A-Based Locus-Specific Multicistronic Expression in Stable Mouse Lines
Source: PLoS One. 2011 Dec 21;6(12):e28885. doi: 10.1371/journal.pone.0028885 (PMC3244433; doi:10.1371/journal.pone.0028885)
Supplement: Table S1 — MFI, percentage of EGFP+ cells and overall EGFP fluorescence of E12.5 Sox9 mouse embryos. Raw values of MFI and percentage of EGFP+ for each Sox9IE/IE and Sox9FE/FE mouse embryo. Overall EGFP fluorescence was calculated by multiplying MFI and percentage of EGFP+ cells. MFI – Mean fluorescence intensity; EGFP – Enhanced green fluorescence protein; SE –Standard error. (DOC) [file pone.0028885.s001.doc]

**Table S1. MFI, percentage of EGFP^+^ cells and overall EGFP fluorescence of E12.5 *Sox9* mouse embryos.**

|  | ***Sox9^IE/IE^*** | | | ***Sox9^FE/FE^*** | | |
| --- | --- | --- | --- | --- | --- | --- |
| **Embryo No.** | **MFI** | **% EGFP^+^ Cells** | **Overall EGFP Fluorescence** | **MFI** | **% EGFP^+^ Cells** | **Overall EGFP Fluorescence** |
| 1 | 3042 | 3.70 | 11255.40 | 4257 | 19.1 | 81308.70 |
| 2 | 3105 | 5.90 | 18319.50 | 4792 | 17.6 | 84339.20 |
| 3 | 3153 | 4.60 | 14503.80 | 4170 | 19.2 | 80064.00 |
| 4 | 3034 | 3.50 | 10619.00 | 4874 | 19.0 | 92606.00 |
| 5 | 3121 | 3.80 | 11859.80 | - | - | - |
| 6 | 3142 | 5.40 | 16966.80 | - | - | - |
| 7 | 3112 | 5.00 | 15560.00 | - | - | - |
| 8 | 2989 | 4.00 | 11956.00 | - | - | - |
| **Mean** | 3087.25 | 4.488 | **13880.04** | 4523.25 | 18.73 | **84579.48** |
| **SE ±** | 20.67 | 0.311 | **1015.55** | 180.49 | 0.377 | **2822.09** |
