# Supplementary material for: Comparison of IRES and F2A-Based Locus-Specific Multicistronic Expression in Stable Mouse Lines
Source: PLoS One. 2011 Dec 21;6(12):e28885. doi: 10.1371/journal.pone.0028885 (PMC3244433; doi:10.1371/journal.pone.0028885)
Supplement: Table S2 — MFI, percentage of EGFP+ cells and overall EGFP fluorescence of E12.5 Bapx1 mouse embryos. Raw values of MFI and percentage of EGFP+ for each Bapx1EGFP/EGFP, Bapx1ICIE/ICIE and Bapx1FCFE/FCFE mouse embryo. Overall EGFP fluorescence was calculated by multiplying MFI and percentage of EGFP+ cells. Percentage difference in deviation of mean overall fluorescence from endogenous levels between F2A and IRES Bapx1 embryos = [(A–B)−(A–C)]/A *100% = 36.5%. MFI – Mean fluorescence intensity; EGFP – Enhanced green fluorescence protein; SE –Standard error. (DOC) [file pone.0028885.s002.doc]

**Table S2. MFI, percentage of EGFP^+^ cells and overall EGFP fluorescence of E12.5 *Bapx1*** mouse embryos.

|  | ***Bapx1^EGFP/EGFP^* (A)** | | | ***Bapx1^ICIE/ICIE^* (B)** | | | ***Bapx1^FCFE/FCFE^* (C)** | | |
| --- | --- | --- | --- | --- | --- | --- | --- | --- | --- |
| **Embryo No.** | **MFI** | **% EGFP^+^ Cells** | **Overall EGFP Fluorescence** | **MFI** | **% EGFP^+^ Cells** | **Overall EGFP Fluorescence** | **MFI** | **% EGFP^+^ Cells** | **Overall EGFP Fluorescence** |
| 1 | 2069 | 1.80 | 3724.20 | 1300 | 1.10 | 1430.00 | 1840 | 2.70 | 4968.00 |
| 2 | 2854 | 2.00 | 5708.00 | 1532 | 1.10 | 1685.20 | 1971 | 1.70 | 3350.70 |
| 3 | 2076 | 1.70 | 3529.20 | 1082 | 1.10 | 1190.20 | 1565 | 1.30 | 2034.50 |
| 4 | 2037 | 2.00 | 4074.00 | 1328 | 1.20 | 1593.60 | 1976 | 2.60 | 5137.60 |
| 5 | 2411 | 1.40 | 3375.40 | 1373 | 1.50 | 2059.50 | 1580 | 2.40 | 3792.00 |
| 6 | 2474 | 1.70 | 4205.80 | 1137 | 1.10 | 1250.70 | 1712 | 1.30 | 2225.60 |
| 7 | 2143 | 2.10 | 4500.30 | 1202 | 0.90 | 1081.80 | 1882 | 1.40 | 2634.80 |
| 8 | - | - | - | 1240 | 0.50 | 620.00 | 1813 | 1.20 | 2175.60 |
| 9 | - | - | - | 666 | 1.70 | 1132.20 | 1565 | 1.20 | 1878.00 |
| 10 | - | - | - | - | - | - | 1421 | 1.30 | 1847.30 |
| 11 | - | - | - | - | - | - | 920 | 1.50 | 1380.00 |
| **Mean** | 2294.86 | 1.81 | **4159.56** | 1206.67 | 1.13 | **1338.13** | 1658.64 | 1.69 | **2856.74** |
| **SE ±** | 114.09 | 0.0911 | **297.81** | 80.86 | 0.113 | **137.83** | 92.12 | 0.176 | **387.58** |
