# Supplementary material for: Comparison of IRES and F2A-Based Locus-Specific Multicistronic Expression in Stable Mouse Lines
Source: PLoS One. 2011 Dec 21;6(12):e28885. doi: 10.1371/journal.pone.0028885 (PMC3244433; doi:10.1371/journal.pone.0028885)
Supplement: Table S3 — Densitometry analysis of the Western blots. Raw densitometry values calculated for the protein bands in the Western blots of figures 2D, 3 and 7. 2A - residual 23 amino acids of F2A; EGFP – Enhanced green fluorescence protein; OD – optical density. (DOC) [file pone.0028885.s003.doc]

**Table S3. Densitometry analysis of the Western blots.**

| **Figure** | **Primary Antibody** | **Lane** | **Protein Bands** | **Density (OD/mm2)** | **Density normalized to histone (OD/mm2)** | **Percentage of fusion protein (%)** |
| --- | --- | --- | --- | --- | --- | --- |
| 2D | anti-Sox9 | *Sox9^FE/FE^* | Sox9-EGFP | 244.26 | 0.618 | 42.2 |
|  |  |  | Sox9-2A | 334.80 | 0.848 | - |
|  |  | *Sox9^IE/IE^* | Sox9 | 400.36 | 1.052 | - |
|  | anti-GFP | *Sox9^FE/FE^* | EGFP | 342.92 | 0.868 | - |
|  |  | *Sox9^IE/IE^* | EGFP | 301.29 | 0.791 | - |
|  | anti-Histone | *Sox9^FE/FE^* | Histone | 395.04 | - | - |
|  |  | *Sox9^IE/IE^* | Histone | 380.70 | - | - |
| 3 | anti-Bapx1 | *FLAG_3_-Bapx1^FE^* | FLAG_3_-Bapx1-EGFP | 29460.07 | - | 49.4 |
|  |  |  | FLAG_3_-Bapx1 | 30145.21 | - | - |
| 7 | anti-Cre | *Bapx1^FCFE/FCFE^* | Cre-EGFP | 250.30 | 0.555 | 55.3 |
|  |  |  | Cre-2A | 202.16 | 0.448 | - |
|  |  | *Bapx1^ICIE/ICIE^* | Cre | 284.92 | 0.680 | - |
|  | anti-GFP | *Bapx1^FCFE/FCFE^* | Cre-EGFP | 305.16 | 0.677 | 62.2 |
|  |  |  | EGFP | 185.42 | 0.411 | - |
|  |  | *Bapx1^ICIE/ICIE^* | EGFP | 210.03 | 0.501 | - |
|  | anti-Histone | *Bapx1^FCFE/FCFE^* | Histone | 450.99 | - | - |
|  |  | *Bapx1^ICIE/ICIE^* | Histone | 419.28 | - | - |
